# Iron Overloading Potentiates the Antitumor Activity of 5-Fluorouracil by Promoting Apoptosis and Ferroptosis in Colorectal Cancer Cells

**DOI:** 10.1007/s12013-024-01463-x

**Published:** 2024-08-04

**Authors:** Bilal Rah, Jasmin Shafarin, Asima Karim, Khuloud Bajbouj, Mawieh Hamad, Jibran Sualeh Muhammad

**Affiliations:** 1 Iron Biology Research Group, Research Institute for Medical and Health Sciences, University of Sharjah, Sharjah, United Arab Emirates; 2 Department of Basic Medical Sciences, College of Medicine, University of Sharjah, Sharjah, United Arab Emirates; 3 Department of Medical Laboratory Sciences, College of Health Sciences, University of Sharjah, Sharjah, United Arab Emirates; 4 Department of Biomedical Sciences, College of Medicine and Health, University of Birmingham, Birmingham, United Kingdom

**Keywords:** Colorectal cancer, Ferroptosis, Chemosensitivity, Ferric ammonium citrate, Apoptosis, Iron overload

## Abstract

Resistance to 5-fluorouracil (5-FU) remains a significant challenge in colorectal cancer (CRC) treatment. Ferric ammonium citrate (FAC) is commonly used as an iron supplement due to its food-fortification properties; however, its potential role as a chemosensitizer in cancer therapy has not been studied. In this study, we explored the ability of FAC to sensitize CRC cells and increase their susceptibility to 5-FU-mediated anticancer effects. We assessed cell viability, cell cycle progression, apoptosis, mitochondrial membrane potential (MMP), reactive oxygen species (ROS) levels, ferroptosis, and iron metabolism-related protein expression using two CRC cell lines. Additionally, we conducted in silico analyses to compare iron markers in normal colon and CRC tumor tissues. Compared to controls, CRC cells pretreated with FAC and then treated with 5-FU exhibited significantly reduced growth and viability, along with increased ROS-mediated ferroptosis. Mechanistically, FAC-pretreated then 5-FU-treated CRC cells showed enhanced apoptosis, increased Bak/Bax expression, MMP depolarization, and decreased antiapoptotic protein levels (Bcl-2 and Bcl-xL). This combined treatment also led to G2/M cell cycle arrest, upregulation of p21 and p27, and downregulation of cyclin D1, c-Myc, survivin, and GPX4. Analysis of human colon tumor tissue revealed decreased expression of *IRP-1*, *HMOX-1*, and *FTH1* but increased *HAMP* expression. In contrast, FAC-pretreated/5-FU-treated CRC cells exhibited a reverse pattern, suggesting that FAC-induced chemosensitization enhances 5-FU-mediated anticancer activity in CRC by disrupting iron homeostasis. These findings highlight the potential of iron overload as a chemosensitization strategy for improving CRC chemotherapy.

## Introduction

Colorectal cancer (CRC) is the third most diagnosed cancer worldwide and the second deadliest cancer after lung cancer. Although significant progress has been achieved in CRC therapy, which involves tumor surgical resection followed by well-planned chemotherapy [1], nearly 50% of CRC patients are at risk of recurrence and metastasis bringing down the five-year survival to only 10% [1]. The drug of choice for curative and palliative therapy in CRC patients is 5-fluorouracil (5-FU), but its response rate for advanced CRC patients is only 10–15%. For advanced CRC patients, 5-FU is utilized in combination with folinic acid and oxaliplatin as first-line chemotherapy; however, besides resistance, the combination significantly increases toxicity, cost, and patient mortality [2]. Therefore, the search for alternative chemosensitizing agents is urgently needed, especially for CRC patients with chemoresistance to the aforementioned therapies.

Ferric ammonium citrate (FAC) or 2-hydroxy-1, 2, 3-propane tricarboxylic acid, a double salt of ammonium citrate and ferric citrate, is commonly used as a food fortifier in various baked goods, dairy products, and microbial cultures. As an iron supplement, FAC is also used as a hematinic agent to treat iron deficiency anemia [3]. Besides governing numerous metabolic processes in various types of cells, iron is essentially required for cellular growth and proliferation by helping the ribonucleotide reductase enzyme to synthesize DNA for neoplastic cells [4]. High intracellular iron levels are known to modulate many oncogenic signaling pathways, including Wnt, JAK/STAT, and p53 in such a way that promotes tumorigenesis [5–7]. Elevated levels of intracellular iron and iron importers are hallmarks in many cancers, by promoting the Warburg effect in CRC cells thereby modulating nuclear factor erythroid 2-related factor (NRF2)-mediated ROS production in favor of tumorigenesis [8]. That said, iron overload also induces oxidative stress leads to lipid peroxidation of the cell membrane, and disrupts several signaling pathways that eventually culminate in ferroptosis and cell death [9].A recent report has suggested that iron in combination with compound Kushen injections promotes chemosensitization of CRC cells by reducing TOP2A and p53 expression [10].

Ferroptosis, a non-apoptotic, iron-regulated cell death, is characterized by the inactivation of antioxidant enzyme GPX4, accumulation of ROS, and induction of lipid peroxidation and consequently cell death [11]. Ferroptosis is very distinct from apoptosis or other forms of cell death as cells show increased membrane density, smaller mitochondria with reduced cristae, and elevated cytoplasmic ROS levels [12]. Ferroptosis has emerged as a promising cell death mechanism for drug-resistant cancers; in the case of CRC, it targets numerous molecules in resistant cells [13]. Although FAC elevates intracellular iron levels and induces oxidative cellular stress in various cellular models of cancer, whether it sensitizes cells to the anti-tumor effects of chemotherapy has yet to be investigated.

In this study, we investigated whether FAC-induced iron overloading could enhance cell death via apoptosis and ferroptosis in 5-FU-treated CRC cells. For the first time, we demonstrated that 5-FU-treatment in FAC-pretreated CRC cells reduced cell viability, induced cell cycle arrest, promoted ROS production, and augmented cell death via apoptosis as well as ferroptosis.

## Materials and Methods

### Materials

Cell culture-tested ferric ammonium citrate (FAC) was purchased from Hi-Media. 5-Florouracil (5-FU), MTT, trypan blue, fetal bovine serum (FBS), RSL3, Ferrostatin (Ferro)-1, Deferoxamine (DFO), carbobenzoxy-valyl-alanyl-aspartyl-[O-methyl]-fluoromethylketone (Z-VAD-FMK), Bradford reagent for protein estimation, and streptomycin-penicillin solution were procured from Sigma-Aldrich (St. Louis, USA). Apoptosis detection annexin V and FITC kit, Propidium iodide (PI) staining kit for cell cycle analysis, and JC-1 staining for mitochondrial membrane potential detection kit were purchased from Abcam. Antibodies such as poly (ADP-ribose) polymerase 1 (PARP1), caspase-3, caspase-9, survivin, cyclin D1, p27, C-Myc, p21, Bcl-xL, Bak, Bax, and Bcl-2 were purchased from cell signal technology, whereas iron metabolism associated antibodies IRP-1, HMOX-1, TFRC, Hepcidin, and FTH1 were purchased from Abcam. Cell culture medium Roswell Park Memorial Institute (RPMI)-1640 was procured from Gibco Life Technologies (Waltham, USA).

### Cell Culture

Human CRC cell lines HCT-116 (RRID: CVCL_0291) and HT-29 (RRID: CVCL_0320) were procured from the American Type Culture Collection (ATCC, Manassas, USA). HCT-116 cell line was isolated from an adult male diagnosed with colorectal cancer, harboring mutations in *KRAS* and *PI3KCA* genes with wildtype *p53*, *BRAF*, and *PTEN* genes, whereas the HT-29 cell line was isolated from an adult white female diagnosed with colorectal adenocarcinoma, harboring mutations in p53 and BRAF genes with wildtype KRAS, BRAF and PTEN genes. Both the cell lines were mycoplasma-free and authenticated using short tandem repeat (STR) genotyping and verified to be identical with the STR profile in comparing databases. Cells were cultured and maintained in RPMI medium which is supplemented with 10% (v/v) FBS and 1% penicillin-streptomycin antibiotic solution to avoid any microbial contamination. The cells were maintained in 5% CO_2_ at 37 °C and periodically checked for mycoplasma contamination. Cell treatment involved the seeding of 5 × 10^3^ or 5 × 10^5^ CRC cells per well using a 96-well plate or a 6-well plate respectively. Cells were allowed to attach to the bottom surface of the well for 24 h. The next day, cells were left untreated or treated with FAC for 24, 48, or 72 hrs. For certain experiments, subsequent treatment with 5-FU of FAC-treated or control cells was performed. Treated cells were cultured for 24 h in a 5% humidified CO_2_ incubator at 37 °C.

### Cell Viability Assay (MTT Assay)

Cell viability was determined by 3-(4,5-dimethylthiazol-2-yl)-2, 5-diphenyltetrazolium bromide (MTT) assay as described previously [14]. Briefly, 5 × 10^3^ CRC cells were plated in each well of the 96-well plate allowing them to be attached to the bottom surface of the well. The next day, cells plated in triplicate were exposed to different concentrations of 5-FU and FAC for 24, 48, and 72 h in a 5% humidified CO_2_ incubator at 37 °C. After the completion of the above-mentioned time points, 5-FU and FAC-exposed cells were incubated with 5 mg/mL MTT dye for the next 3–4 h at 37 °C. Next, the MTT solution was removed and 150 µL of DMSO was added to each well to dissolve formazan crystals formed during the incubation of MTT dye. The absorbance of the deep blue to a pink color solution developed with DMSO was measured at 570 nm using a plate reader. The percentage of cell viability of cells exposed to various concentrations of 5-FU and FAC was determined as per the protocol described previously.

### Cell Viability (Trypan Blue Exclusion Assay)

Cell viability of CRC cells was determined by trypan blue exclusion assay. Briefly, 5 × 10^3^ seeded cells were treated with varying concentrations of 5-FU, FAC, and FAC-pretreated/5-FU-treated CRC cells for different time points. After the required time points cells were harvested, collected, and resuspended in fresh medium or PBS. Resuspended cells were diluted and mixed in 1:1 (v/v) trypan blue (0.4%) and were loaded (10 µL) on a hemocytometer to count the viable cells. Owing to its high molecular weight, trypan blue is unable to enter the live cells through intact cell membrane, however, dead cells have disrupted cell membrane, which eventually allows trypan blue to enter the cells thereby stained blue in a transparent background. Cells were counted by cell counter and the percentage of cell viability of cells exposed to various 5-FU and FAC was determined as per the protocol previously [15].

### Phase Contrast Microscopy

For phase-contrast microscopy, 5 × 10^5^ HCT-116 or HT-29 cells were plated in 30 mm Petri dishes and allowed to adhere to the bottom surface of the petri dish overnight. The next day, cells were exposed to varying concentrations of 5-FU and FAC alone or in FAC-pretreated/5-FU-treated cells in addition to the untreated control for 24 h. After the completion of the time point, cells were analyzed both at low (10X) and high (20X) magnification under the phase-contrast microscope to analyze morphological changes in treated cells compared to the untreated control.

### Immunoblotting

HCT-116 cells were seeded at a density of 5 × 10^5^ in a 6-well plate and allowed to adhere overnight. The next morning, adhered cells in a 6-well plate were pretreated with FAC for 24 h, followed by treatment with 5-FU for 24 h alone or FAC-pretreated/5-FU-treated cells in addition to the untreated control. Treated cells were harvested and processed for cell lysis in RIPA buffer. Cell lysis solution was centrifuged (refrigerated) at 12,000 g for 10 min, and the supernatant thus obtained was collected and subjected to protein estimation by the commonly used Bradford method. A total of 20 µg of protein from each treatment was mixed with sample loading buffer, denatured, and loaded into each well of SDS-PAGE gel. After resolving the cell lysate in SDS-PAGE gel properly, the resolved proteins were subjected to transfer onto the charged PVDF membrane and then incubated in a 5% blocking solution (skimmed milk) for 30 min to avoid any nonspecific binding of the primary antibody. Membranes were then incubated with the primary antibody for either 3 h at room temperature or overnight at 4 °C. Next, the PVDF membrane was washed with TBST buffer three times (10 min each) and incubated with species-specific secondary antibodies for 1 h at room temperature. After incubation of the secondary antibody, the PVDF membrane was washed again with TBST buffer three times (10 min each) and incubated in an enhanced chemiluminescence (ECL) solution for 5 min and the chemiluminescence signal was captured by using chemiluminescence gel doc.

### Cell Cycle Analysis

HCT-116 cells were seeded at a density of 5 × 10^5^ in each well of the 6-well plate and allowed to adhere overnight at 37 °C in a 5% humidified CO_2_ incubator. The next morning, adhered cells plated in a 6-well plate were subjected to treatment with FAC and 5-FU alone and FAC-pretreated/5-FU-treated cells, along with untreated control, for 24 h. After, 24 h treatment cells from each well were harvested, washed with PBS, and fixed for at least 30 min or overnight with 70% ice-cold ethanol. Next, the fixed cells were subjected to RNase (100 µg/mL) at 37 °C for 30 min, followed by propidium iodide (PI) (50 µg/mL) staining in the dark for the next 30 min at 4 °C. The stained cells were analyzed for cell cycle analysis using BD Aria II flow cytometry to observe any change in cell cycle phases after FAC and 5-FU treatment.

### Apoptotic Analysis

Detection and quantification of apoptotic cells were analyzed by using Annexin V and FITC apoptosis detection kit. As per the manufacturer’s instructions, 5 × 10^5^ cells were seeded in each well of a 6-well plate and allowed to adhere to the bottom surface of the well overnight at 37 °C. The next morning, cells were exposed to FAC and 5-FU alone or FAC-pretreated/5-FU-treated cells, along with untreated control, for 24 h. Treated cells were harvested, washed with PBS, and subjected to Annexin V FITC/PI staining as per manufacturer protocol and were analyzed using BD Aria II flow cytometry.

### Detection of Reactive Oxygen Species

Detection and quantification of ROS using DCFH-DA dye as described previously [16]. A stable non-fluorescent compound, DCFH-DA, is a permeable compound that, upon entering the cell, is further oxidized by ROS to form the fluorescent compound DCF, which is stable for a few hours. To determine and quantify ROS, 5 × 10^5^ cells were plated in each well of 6-well plates. Upon treatment with FAC and 5-FU alone or FAC-pretreated/5-FU-treated cells in addition to untreated control for 24 h, cells were harvested and incubated with DCFH-DA (10 µM) at 37 °C for 30 min in the dark. Next cells were washed and resuspended in PBS and simultaneously kept on ice to proceed for immediate analysis of ROS quantification by BD Aria II flow cytometry.

### Depolarization of Mitochondrial Membrane Potential (MMP)

Determination of the depolarization of MMP is an important analysis to discriminate between intrinsic and extrinsic apoptosis cell populations. For the measurement of depolarization of MMP, HCT-116 cells were seeded at a density of 5 × 10^5^ cells in each well of a 6-well plate. After being properly attached to the bottom surface of the well, cells were exposed to FAC and 5-FU alone or FAC-pretreated/5-FU-treated cells in addition to untreated control cells for 24 h. Cells after treatment were harvested, washed with PBS, and incubated with JC-1 dye as per the manufacturer’s instructions in the dark for 15 min at room temperature. Stained cells were simultaneously acquired using BD Aria II flow cytometry for analysis.

### Determination of Intracellular GSH and MDA Levels

Determination of GSH and MDA levels in CRC cells were determined by colorimetric assays. For the measurement of GSH and MDA levels, HCT-116 and HT-29 at a density of 2 × 10^6^ cells in 60 mm petri dishes. After being properly attached to the bottom surface of the petri dish, cells were exposed to FAC for 24 h, followed by treatment with 5-FU for 24 h alone or FAC-pretreated/5-FU-treated cells in presence of ferroptosis inducer RSL3, iron chelator DFO, ferroptosis inhibitor Ferro-1 and apoptosis inhibitor Z-VAD-FMK addition to the untreated control. Cells after treatment were harvested, washed with PBS and processed for homogenization and sonication respective GSH and MDA assay buffers on ice and later centrifuged at 16,000 g for 15 minutes at 4 °C to collect supernatant, The measurement of GSH and MDA activity was performed by taking 100 uL of each processed samples as per the manufacturer’s protocol and absorbance were recorded at 405 and 410 nm, respectively by multiplate reader and compiled as bar diagram.

### Determination of Intracellular Iron Content

Iron assay was used to determine the intracellular iron concentration in CRC cells after indicated treatment. Briefly, HCT-116 and HT-29 cells at a density 2 × 10^6^ cells in 60 mm petri dishes. After being properly attached to the bottom surface of the petri dish, cells were exposed to FAC for 24 h, followed by treatment with 5-FU for 24 h alone or FAC-pretreated/5-FU-treated cells in presence of ferroptosis inducer RSL3, iron chelator DFO, ferroptosis inhibitor Ferro-1 and apoptosis inhibitor Z-VAD-FMK addition to the untreated control. Cells after treatment were harvested, washed with PBS and processed for the cell lysis using homogenization, sonication and then centrifuged at 16,000 g for 15 min at 4 °C to collect supernatant. As per manufacturer’s protocol the each lysate mixed with reagents was incubated in dark at 30 min and absorbance was measured at 593 nm by multiplate reader and compiled as bar diagram.

### In Silico Analysis

For expression analysis of the candidate iron metabolism-related genes in human colon cancer tissues versus normal colon tissues, the cancer genome atlas (TCGA) online portal (https://portal.gdc.cancer.gov/) was accessed. Data is presented as a Box-Whisker plot, showing gene expression levels in normal (n = 41) versus primary colon tumor (n = 286) samples.

### Statistical Analysis

Cell viability and cell growth data as well as protein and gene expression data is presented here as the mean ± standard error of results obtained by at least three independent experiments; statistical analysis was performed using one-way ANOVA. A two-sided value equal to or less than p-value < 0.01 was considered a significant value in all the in vitro experiments. For in silico analysis, the significance of differences between the two samples (Normal versus tumor) was evaluated by using the student’s t-test. Statistical analysis was performed using GraphPad Prism software version 8, and a p-value of <0.01 was considered significant.

## Results

### Effects of 5-FU and FAC on CRC Cell Growth and Viability

**Fig. 1:**
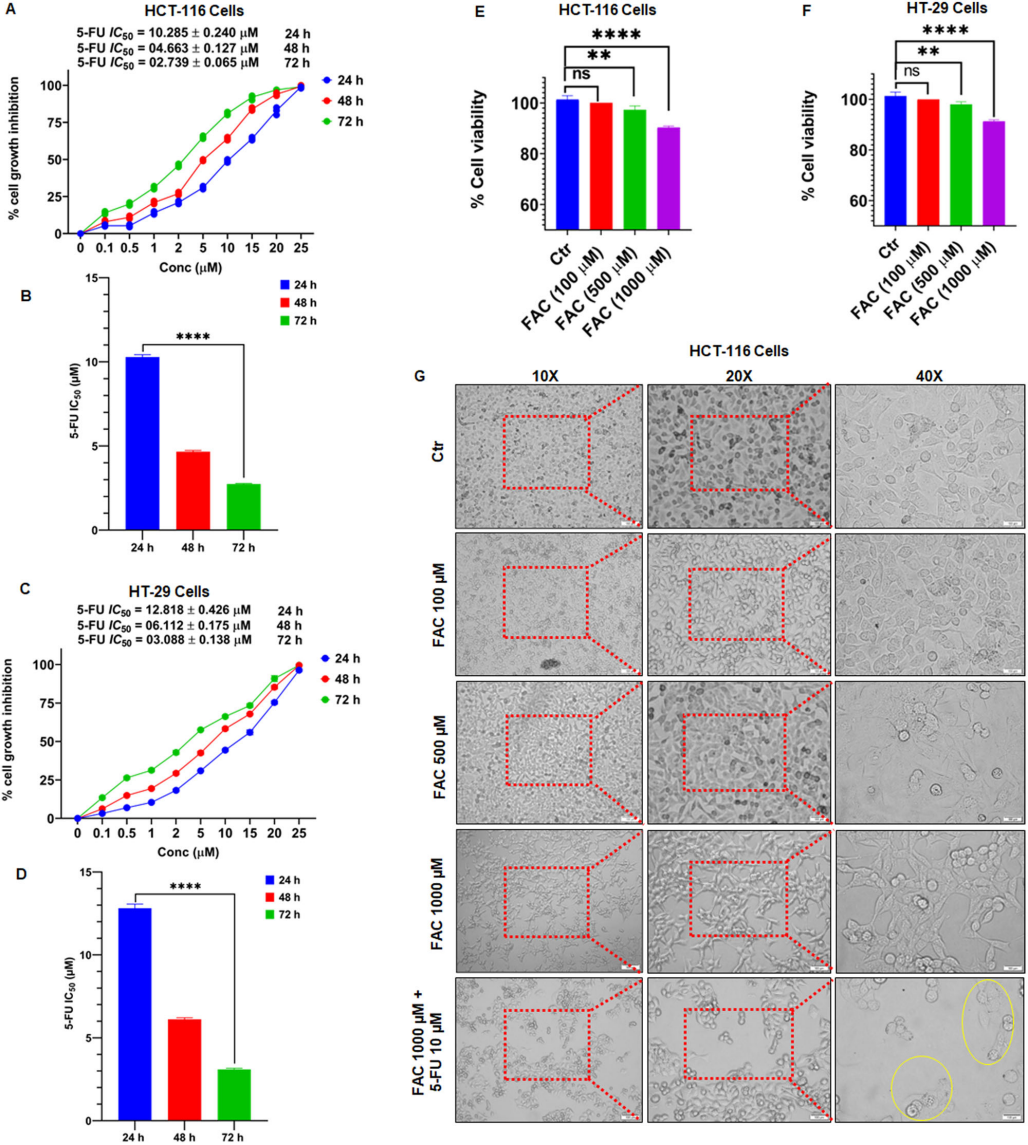
Antiproliferative effects of 5-FU and FAC in HCT-116 and HT-29 cells. Dose and time-dependent cell viability in 5-FU-treated (**A**) HCT-116 and (**C**) HT-29 cells at 24, 48, and 72 h. Bar diagram of 5-FU IC50 values at 24, 48, and 72 h in (**B**) HCT-116 and (**D**) HT-29 cells. Cell viability of (**E**) HCT-116 and (**F**) HT-29 cells treated with 100, 500, or 1000 μM FAC for 24 h. **G** Representative phase-contrast micrographs taken at 10X, 20X, and 40X of HCT-116 treated with 5-FU, FAC for 24 h. Data represent the mean value ± SE of at least three independent experiments. A significant change from the control is indicated by *p ≤ 0.05, **p ≤ 0.01 and ****p ≤ 0.0001

To evaluate the cytotoxic effect of 5-FU and FAC, cells (HCT-116 and HT-29) plated in 96-well plates, were incubated with varying doses of 5-FU ranging from 0.1 to 25 µM for different time points (24, 48, and 72 h). As shown in Fig. 1A–D, 5-FU significantly reduced cell proliferation of HCT-116 and HT-29 cells in a dose- and time-dependent manner. The 50% inhibitory concentration (IC_50_) of 5-FU against HCT-116 cells were 10.285 ± 0.240, 04.663 ± 0.127, 02.739 ± 0.065 µM at 24, 48, and 72 h times points, respectively; 5-FU IC_50_ value in HT-29 cells were 12.818 ± 0.426, 06.112 ± 0.175, and 03.088 ± 0.138 µM at 24, 48, and 72 h time points, respectively. Moreover, cells were plated at a density of 5 × 10^3^ to determine cell viability of different concentrations of FAC ranging from 10–1000 µM for 24 h. Interestingly, we observed less than or equal to 10% antiproliferative effect in HCT-116 and HT-29 at the highest FAC concentration (1000 µM), relative to untreated controls after 24 h of treatment (Fig. 1E, F and Supplementary Fig. 1A, B). By phase-contrast microscopy, we observed a significantly higher number of round dead, floating cells when treated with 5-FU (10 µM); only negligible changes were observed in cells treated with FAC (1000 µM) and in control untreated cells at 24 h post-treatment (Fig. 1G). These findings suggest that 5-FU exerts significant antiproliferative and cytotoxic effects in CRC cells as compared to FAC.

### FAC Pretreatment Sensitizes CRC Cells to the Anti-Growth Activity of 5-FU

**Fig. 2:**
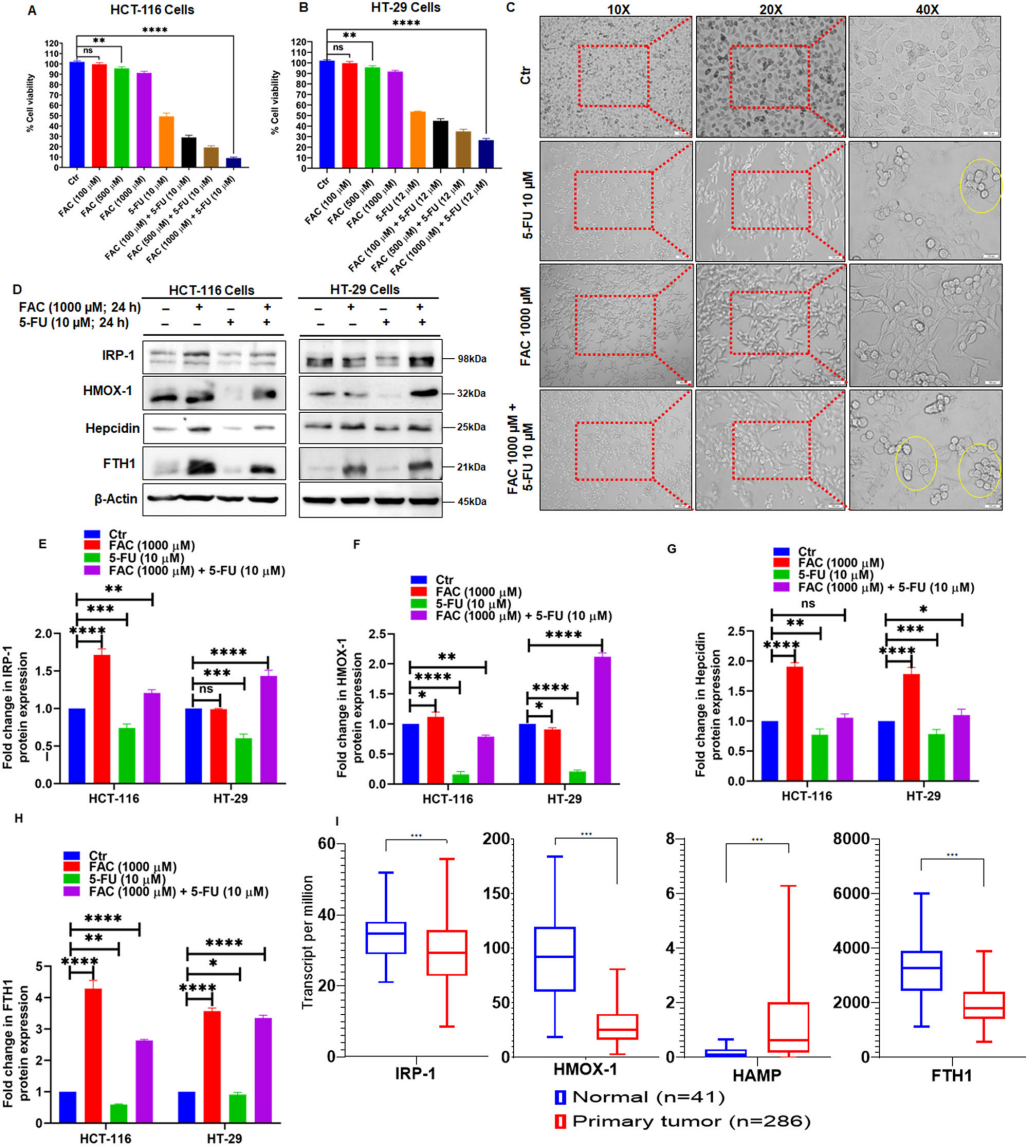
A combination of FAC and 5-FU potentiates the antiproliferative effect in HCT-116 and HT-29 cells. Cell viability determined by MTT assay in (**A**) HCT-116 and (**B**) HT-29 cells exposed to FAC (100, 500, 1000 μM) alone, FAC (100, 500, 1000 μM) + 5-FU (10 μM), 5-FU (10 μM) alone for 24 h. **C** Representative phase-contrast micrographs taken at 10X, 20X, and 40X of HCT-116 treated with FAC (1000 μM), 5-FU (10 μM), (FAC 1000 μM + 5-FU) for 24 h. **D** Expression status of iron regulatory proteins IRP-1, HMOX-1, hepcidin, and FTH-1 in HCT-116 cell lysates treated with FAC (1000 μM), FAC (1000 μM) + 5-FU(10 μM), 5-FU (10 μM) for 24 h. Fold change in the expression of (**E**) IRP-1, (**F**) HMOX-1, (**G**) hepcidin, and (**H**) FTH-1 protein in HCT-116 cells treated with FAC (1000 μM), FAC (1000 μM) + 5-FU(10 μM), 5-FU (10 μM) for 24 h. (**I**) Analysis of TCGA database for the expression of IRP-1, HMOX-1, FTH1, and HAMP in primary colon adenocarcinoma. Data represents the mean value ± SE of at least three independent experiments. Significant change from the control is indicated by *p ≤ 0.05, **p ≤ 0.01 and ****p ≤ 0.0001

To further investigate the antiproliferative effects of FAC pretreatment, HCT-116, and HT-29 cells were treated with 100, 500, and 1000 µM of FAC for 24 h and left alone or further treated with 5-FU (10 µM). Cell viability data (MTT assay and trypan blue exclusion assay) showed no significant antiproliferative effect induced by FAC alone treatment in either HCT-116 or HT-29 cells at 24 h and irrespective of concentration (Fig. 2A, B and Supplementary Fig. 2A, B). In contrast, FAC pretreatment (100, 500, and 1000 µM) for 24 h followed by 5-FU (10 µM) treatment for another 24 h resulted in a significant dose-dependent reduction in cell growth and viability; 9% viability at FAC 1000 µM + 5-FU 10 µM as compared to 49% viability at 5-FU 10 µM. Cell viability in FAC-pretreated-5-FU-treated cells was more pronounced in HCT-116 cells relative to HT-29 cells; 9% viability at FAC 1000 µM / 5-FU 10 µM vs. 26% cell viability at FAC 1000 µM / 5-FU 10 µM (Fig. 2A, B). Phase-contrast microscopy of cells pretreated with FAC (100, 500, and 1000 µM) for 24 h followed by 5-FU (10 µM) for another 24 h showed significantly higher numbers of rounded, floating dead cells relative to controls (Fig. 2C).

To further understand this sensitization effect of FAC pretreatment on the efficacy of 5-FU, the expression of key iron regulatory proteins in FAC-pretreated HCT-116 cells (1000 µM for 24 h) that were subsequently left untreated or treated with 5-FU (10 µM) for another 24 h. A significant increase in the expression of the iron regulatory protein (IRP)-1, heme-oxygenase-1 (HMOX-1), and ferritin heavy chain (FTH1) was noticed in both cells exposed to FAC alone and FAC-pretreated/5-FU-treated cells relative to 5-FU alone treated and untreated controls (Fig. 2D–H). Additionally, upregulation in the expression of hepcidin in cells treated with FAC alone and in FAC-pretreated/5-FU-treated CRC cells relative to 5-FU alone treated and untreated controls were also observed. In silico analysis further supported these observations; in that, the expression of *IRP-1*, *HMOX-1*, and *FTH1* expression was significantly decreased while that of *HAMP* (Hepcidin) was significantly increased in primary colon tumor tissue compared to normal tissue (Fig. 2I). To evaluate whether cell death in FAC-pretreated/5-FU treated cells is p53-dependent, cell viability of HT-29 (p53 mutant) cells treated with FAC, 5-FU, both or neither was evaluated at 24 h post-treatment. As shown in Fig. 2B, the percentage of cell viability in p53 mutant cells was similar to that of the p53 wild-type (HCT-116) cells. Additionally, the pattern of expression of iron regulatory proteins IRP-1, HMOX-1, FTH1, and hepcidin was also similar in both types of cells (Fig. 2D–H). Together, these findings documented the induction of iron overload in FAC-treated cells and showed that disruption of cellular iron metabolism (accumulation of intracellular iron) leads to oxidative stress, which potentiates 5-FU-induced antiproliferative effects irrespective of p53 status in CRC cells.

### FAC Pretreatment Potentiates 5-FU-Mediated Cell Death via Apoptosis and Necroptosis

**Fig. 3:**
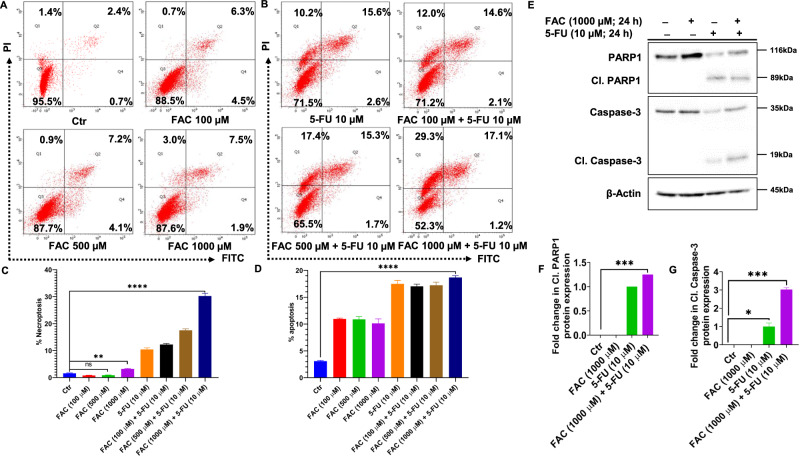
FAC pretreatment potentiates 5-FU-mediated cell death via apoptosis and necroptosis in HCT-116 cells. Quantification of apoptotic, necrotic, and normal cells from four different quadrants by Annexin V FITC and PI staining using flowcytometry in HCT-116 cells treated with (**A**) FAC (100, 500, 1000 μM) for 24 h or (**B**) FAC (100 μM) + 5-FU (10 μM), FAC (500 μM) + 5-FU (10 μM), FAC (1000 μM) + 5-FU (10 μM), 5-FU (10 μM) for 24 h. Bar diagram showing quantification of apoptotic and necroptotic cells in quadrants of HCT-116 cells treated with (**C**) FAC (100, 500, 1000 μM) for 24 h, (**D**) FAC (100 μM) + 5-FU (10 μM), FAC (500 μM) + 5-FU (10 μM), FAC (1000 μM) + 5-FU (10 μM), 5-FU (10 μM) for 24 h. **E** Immunoblotting of PARP1, caspase-3, and their respective cleaved products of HCT-116 cells treated with FAC (1000 μM), 5-FU (10 μM), FAC (1000 μM) + 5-FU(10 µM) for 24 h. Fold change in the expression protein level of (**F**) cleaved product of PARP1 and (**G**) cleaved product of caspase-3 in HCT-116 cells treated with FAC (1000 μM), FAC (1000 μM) + 5-FU (10 µM), 5-FU (10 μM) for 24 h. Data represent the mean value ± SE of at least three independent experiments. Significant change from the control is indicated by *p ≤ 0.05, **p ≤ 0.01 and ****p ≤ 0.0001

To further investigate the after-effects of iron overloading in the form of FAC-pretreated/5-FU-treated CRC cells, we performed an Annexin V-FITC assay for the quantification of apoptotic cells. After FAC pretreatment with different concentrations (100, 500, and 1000 µM), FAC-pretreated/5-FU-treated cells along with 5-FU (10 µM) alone and untreated control for 24 h. As shown in (Fig. 3A–D) a significant increase in apoptotic cell population was observed in FAC-pretreated/5-FU-treated (18.3% at FAC 1000 µM + 5-FU 10 µM) cells, followed by 5-FU (10 µM) (18.2%) when compared with FAC alone (9.4% at FAC 1000 µM) and untreated control (3.1%). However, we did not find a marked difference in apoptotic cell populations between the FAC-pretreated/5-FU (18.3%) treatment and the 5-FU treatment alone (18.2%). However, a sharp increase in the necroptotic population of cells was noticed in cells initially exposed to FAC-pretreated/5-FU treatment (29.3% at FAC 1000 µM + 5-FU 10 µM) than FAC (3% at FAC 1000 µM), 5-FU (10.2% at 5-FU 10 µM) alone, and untreated control (1.4%) cells. Additionally, immunoblotting results of cell lysates of cells exposed to FAC concentration (1000 µM) alone, FAC-pretreated/5-FU-treated cells along with 5-FU (10 µM) alone and untreated control for 24 h, revealed that cleavage products of PARP1 and caspase-3 were detected in lanes loaded with cell lysates treated with 5-FU alone and FAC-pretreated/5-FU-treated cells when compared with FAC alone and untreated control cell lysates (Fig. 3E–G). However, the detected cleaved PARP1 and caspase-3 products are more prominent in FAC-pretreated/5-FU-treated CRC cells than 5-FU alone treated ones, indicating that more cell death is triggered in these cells. Together, these results demonstrated that besides inducing apoptosis, iron overload pushes a significant population of FAC-pretreated/5-FU-treated cells towards necroptosis cell death mechanisms, which need to be investigated in the next experiments to determine whether the necroptotic population quantified in the Q1 quadrant is necroptosis- or ferroptosis-induced cell death.

### FAC Pretreatment Depolarizes Mitochondrial Membrane Potential (MMP)

**Fig. 4:**
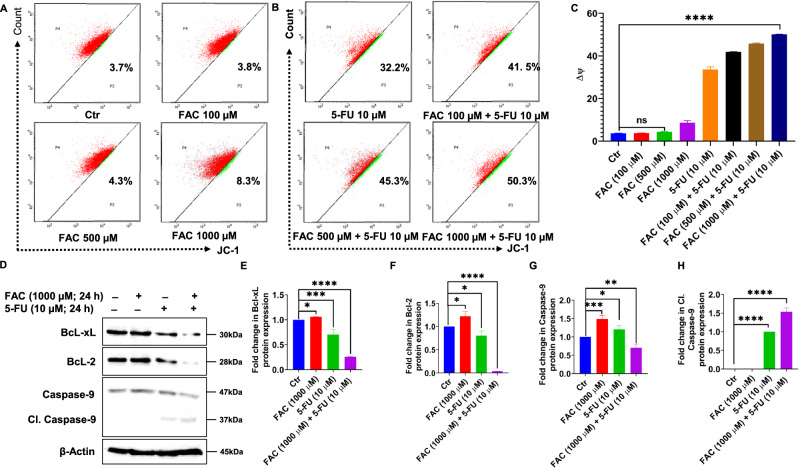
Combination (FAC + 5-FU) treatment depolarizes mitochondrial membrane potential (MMP) in HCT-116 cells. Quantification of depolarization of mitochondrial membrane potential and normal cells by JC-1 staining using flow cytometry in HCT-116 cells treated with (**A**) FAC (100, 500, 1000 μM) for 24 h or (**B**) FAC (1000 μM), FAC (100, 500, 1000 μM) + 5-FU (10 μM), 5-FU (10 μM) for 24 h. **C** Bar diagram showing quantification of JC-1 positive staining of HCT-116 cells treated with FAC (100, 500, 1000 μM) alone or in a combination of FAC (100, 500, 1000 μM) plus 5-FU (10 μM), 5-FU (10 μM) for 24 h. **D** Immunoblotting of proapoptotic caspase-9 and antiapoptotic proteins Bcl-2, Bcl-xL expression status in the cell lysates of HCT-116 cells treated with FAC (1000 μM), 5-FU (10 μM), (FAC (1000 μM) plus+ 5-FU(10 µM) for 24 h. Fold change in the expression of (**E**) Bcl-xL, (**F**) Bcl-2, (**G**) caspase-9 or (**H**) Cleaved caspase-9 protein in HCT-116 cells treated with FAC (1000 μM), FAC (1000 μM) plus+ 5-FU(10 µM), 5-FU (10 μM) for 24 h. Data represent the mean value ± SE of at least three independent experiments; Significant change from the control is indicated by *p ≤ 0.05, **p ≤ 0.01, and ****p ≤ 0.0001

To verify whether the FAC-pretreated/5-FU-treated CRC cells are activating the mitochondrial-dependent apoptotic pathway, we performed a mitochondrial membrane potential (MMP) assay to determine the depolarization of MMP in CRC cells pretreated with different concentrations of FAC (100, 500, and 1000 µM) alone, FAC-pretreated/5-FU-treated CRC cells along with 5-FU (10 µM) alone, and untreated control for 24 h. As shown in Fig. 4A–C a significant increase in MMP depolarization was observed by flowcytometry in FAC-pretreated/5-FU-treated CRC cells as well as in 5-FU alone treated cells when compared with FAC alone and untreated control-treated CRC cells. Furthermore, by immunoblotting analysis, we noticed a drastic reduction in Bcl-2 and Bcl-xL protein expression in cell lysates of FAC-pretreated/5-FU-treated cells compared to the untreated control (Fig. 4D–F). Additionally, caspase-9 which is reported to play a key role in intrinsic apoptosis has been activated by the release of cleaved caspase-3 product after FAC-pretreated/5-FU-treated CRC cells, and 5-FU alone compared to untreated control and FAC alone treatment (Fig. 4D, G, H). Together, these results suggest that iron overloading in the form of FAC chemosensitizes CRC cells and drives greater numbers of cells toward apoptosis.

### FAC and 5-FU Combination Inhibits the Cell Cycle at the G2/M Phase

**Fig. 5:**
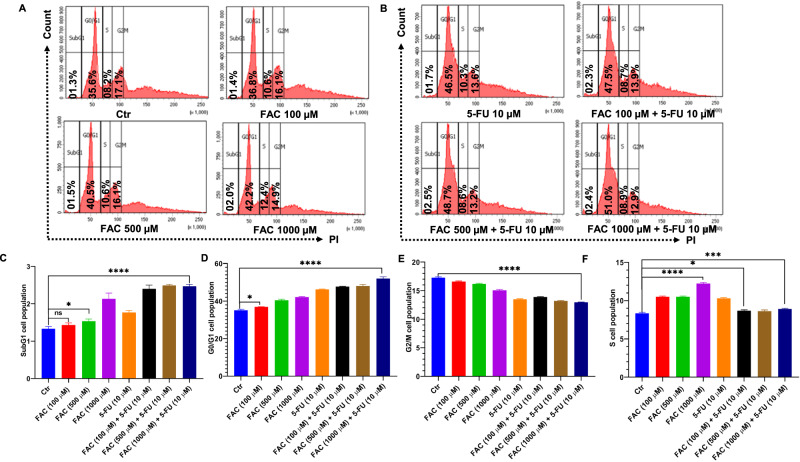
FAC pretreatment and 5-FU treatment trigger a G2/M cell cycle arrest in HCT-116 cells. Cell cycle analysis by PI staining using flow cytometry in HCT-116 cells treated with (**A**) FAC (100, 500, 1000 μM) for 24 h, (**B**) FAC (100, 500, 1000 μM) + 5-FU (10 μM), 5-FU (10 μM) for 24 h. The bar diagram represents the quantification of (**C**) subG1, (**D**) G0-G1, (**E**) G2-M, and (**F**) S cells following treatment with FAC (100, 500, 1000 μM), FAC (100, 500, 1000 μM) + 5-FU (10 μM), 5-FU (10 μM) and no treatment for 24 h. Data represent the mean value ± SE of at least three independent experiments; Significant change from the control is indicated by *p ≤ 0.05, **p ≤ 0.01, and ****p ≤ 0.0001

We next examine whether FAC-pretreated/5-FU-treated CRC cells could inhibit the cell cycle to attenuate cellular growth in CRC cells. To do that, cells were treated with different concentrations of FAC (100, 500, and 1000 µM) alone, and FAC-pretreated/5-FU-treated CRC cells along with 5-FU (10 µM) alone and untreated control for 24 h. As shown in Fig. 5A, B, our cell cycle analysis by flow cytometry revealed that FAC-pretreated/5-FU-treated cells as well as 5-FU alone treated cells could not progress towards the G2/M phase to enter the mitosis phase of the cell cycle, which leads to significant inhibition of cellular growth in CRC cells compared to FAC alone and untreated control cells (Fig. 5C–F).

### FAC and 5-FU Combination Modulates Key Proteins of the Cell Cycle in CRC Cells

**Fig. 6:**
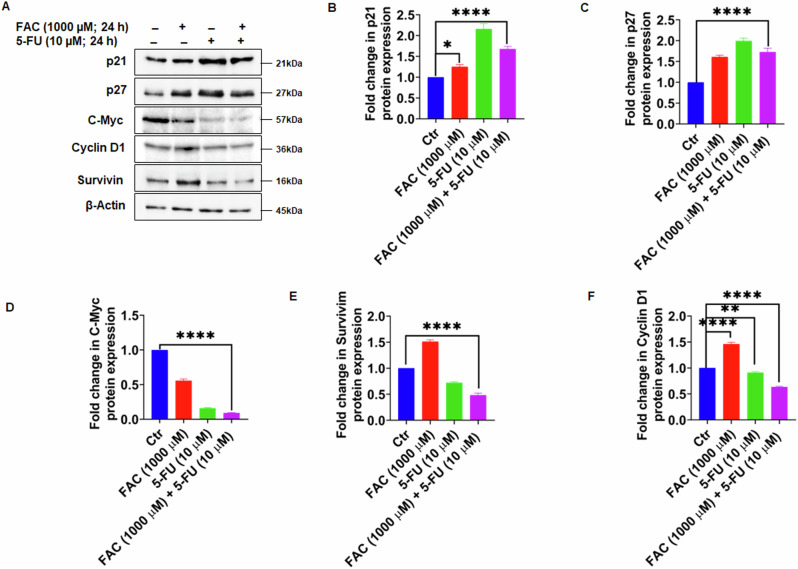
FAC + 5-FU treatment modulates cell cycle regulatory and checkpoint proteins in HCT-116 cells. **A** Expression of checkpoint proteins (p21 and p27) and cell cycle regulatory proteins (cyclin D1, C-Myc, and survivin) in lysates obtained from CRC cells treated with FAC (1000 μM), 5-FU (10 μM), (FAC (1000 μM) + 5-FU (10 μM) and untreated cells left untreated for 24 h. Fold change in the expression of (**B**) checkpoint protein p21, (**C**) p27 (**D**) c-Myc (**E**) survivin, and (**F**) cyclin D1 in lysates obtained from CRC cells treated with FAC (1000 μM), 5-FU (10 μM), (FAC (1000 μM) + 5-FU (10 μM) for 24 h and untreated cells. Data represents the mean value ± SE of at least three independent experiments. Significant change from the control is indicated by *p ≤ 0.05, **p ≤ 0.01 and ****p ≤ 0.0001

Further, our immunoblotting results of cell lysates exposed to FAC-pretreated/5-FU-treated cells, along with 5-FU, FAC, and untreated control cells for 24 h, revealed a marked reduction in the protein expression of cell cycle regulatory proteins C-Myc, cyclin D1, and survivin with a concomitant increase in the protein expression of p21 and p27, which act as checkpoint regulators and play a critical role in regulating cell cycle (Fig. 6A). The effect of induction of cell cycle checkpoints p21 and p27 is more pronounced in 5-FU alone treated cells than in FAC-pretreated/5-FU-treated CRC cells (Fig. 6B, C), however, the decrease in protein expression of C-Myc, cyclin D1, and survivin is more in FAC-pretreated/5-FU-treated CRC cells than in 5-FU treated CRC cells (Fig. 6D–E). In summary, these results demonstrated that FAC-pretreated/5-FU-treated cells and 5-FU alone treatment have a similar effect concerning cell cycle arrest in CRC cells and attenuate the G2/M phase of the cell cycle.

### FAC and 5-FU Combination Triggered Reactive Oxygen Species (ROS)-Mediated Ferroptosis

**Fig. 7:**
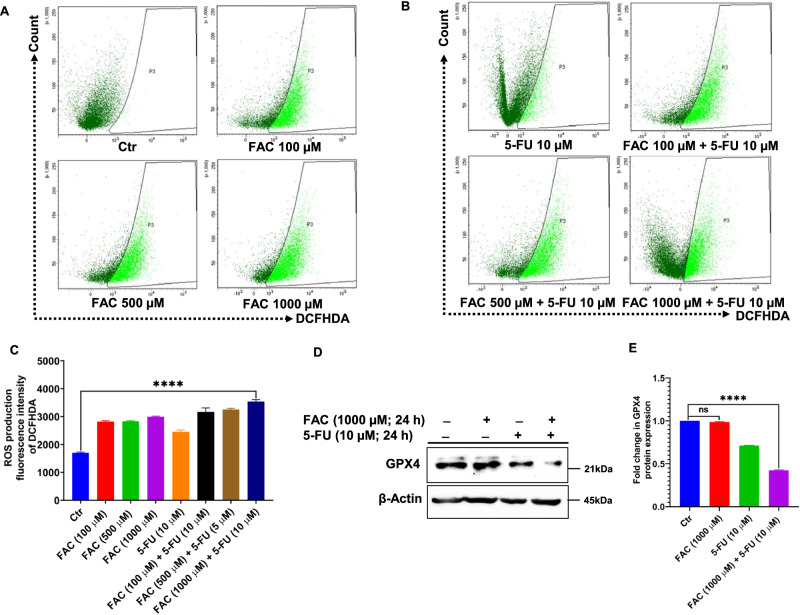
FAC + 5-FU treatment induces oxidative stress-mediated reactive oxygen species (ROS) and ferroptosis in HCT-116 cells. ROS production in CRC cells treated with (**A**) FAC (100, 500, 1000 μM) alone or (**B**) FAC (100, 500, 1000 μM) + 5-FU (10 μM), 5-FU (10 μM) for 24 h; ROS production was determined by the DCFHDA staining flow cytometry-based method. **C** Bar diagram representing DCFHDA staining intensity based on 3 independent experiments as in (**A**) and (**B**). (**D**) Expression of GPX4 in lysates obtained from CRC cells treated with FAC (1000 μM), 5-FU (10 μM), (FAC (1000 μM) + 5-FU (10 μM) for 24 h, and untreated cells. **E** Fold change in GPX4 expression based on three independent experiments as in D. Significant change from the control is indicated by *p ≤ 0.05, **p ≤ 0.01 and ****p ≤ 0.0001

Next, we sought to determine whether FAC-pretreated/5-FU-treated CRC cells could trigger oxidative stress-mediated ROS production. To evaluate the quantification of ROS, CRC cells were treated for 24 h with different concentrations of FAC (100, 500, and 1000 µM) alone, FAC-pretreated/5-FU-treated along with 5-FU (10 µM) alone, and untreated control. As shown in Fig. 7A, B quantification of DCFHDA stained cells by flow cytometry was performed. Our results showed that cells exposed to FAC-pretreated/5-FU-treated cells have a dose-dependent increase in DCFHDA fluorescence compared to 5-FU, whereas in FAC alone treated cells we noticed a similar trend in the increase in ROS as that of FAC-pretreated/5-FU-treated cells when compared to control untreated Cells (Fig. 7C). However, we observed relatively more DCFHDA fluorescence in FAC alone than in 5-FU, this could be due to iron overload in the form of FAC, which induces oxidative stress that eventually triggers ROS generation in these cells compared to 5-FU alone, and the untreated cells. Furthermore, our immunoblotting results revealed a significant downregulation of the antioxidant protein GPX4 expression in cell lysates of FAC-pretreated/5-FU-treated cells compared to FAC alone, 5-FU alone, and untreated control (Fig. 7D, E). Collectively, these results suggest that iron overload induces oxidative stress in cells as evidenced by ROS production, however, FAC-pretreated/5-FU-treated cells not only induce apoptosis but also trigger more ROS production, which eventually drives oxidative stress in CRC cells toward ferroptosis-mediated cell death.

### FAC and 5-FU Combination Promotes Both Apoptosis and Ferroptosis in CRC Cells

**Fig. 8:**
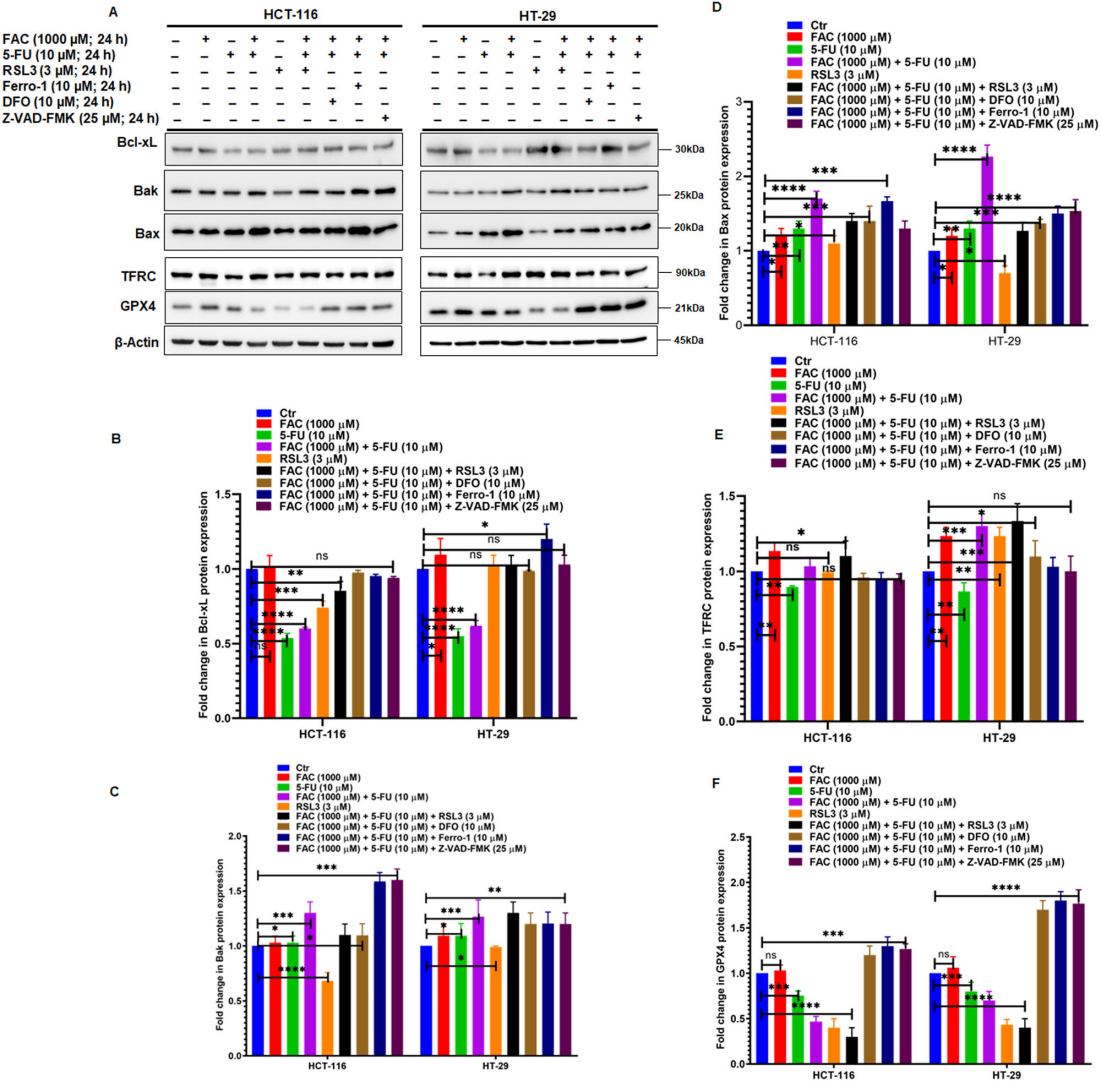
FAC and 5-FU combination promotes both apoptosis and ferroptosis in CRC cells. **A** Expression of antiapoptotic (Bcl-xL) and apoptotic proteins (Bak and Bax) and ferroptosis associated proteins (GPX4, and TFRC) in lysates obtained from CRC cells (HCT-116 and HT-29) treated with FAC (1000 μM), 5-FU (10 μM), (FAC (1000 μM) + 5-FU (10 μM), RSL3, RSL3 + (FAC (1000 μM) + 5-FU (10 μM), DFO + (FAC (1000 μM) + 5-FU (10 μM), Ferro-1 + (FAC (1000 μM) + 5-FU (10 μM), Z-VAD-FMK + (FAC (1000 μM) + 5-FU (10 μM), and left untreated for 24 h. Fold change in the expression of (**B**) Bcl-xL, (**C**) Bak (**D**) Bax (**E**) TFRC, and (**F**) GPX4 in cell lysates obtained from CRC (HCT-116 and HT-29) cells treated with FAC (1000 μM), 5-FU (10 μM), (FAC (1000 μM) + 5-FU (10 μM), RSL3, RSL3 + (FAC (1000 μM) + 5-FU (10 μM), DFO + (FAC (1000 μM) + 5-FU (10 μM), Ferro-1 + (FAC (1000 μM) + 5-FU (10 μM), Z-VAD-FMK + (FAC (1000 μM) + 5-FU (10 μM), and left untreated for 24 h. Data represents the mean value ± SE of at least three independent experiments. Significant change from the control is indicated by *p ≤ 0.05, **p ≤ 0.01 and ****p ≤ 0.0001

**Fig. 9:**
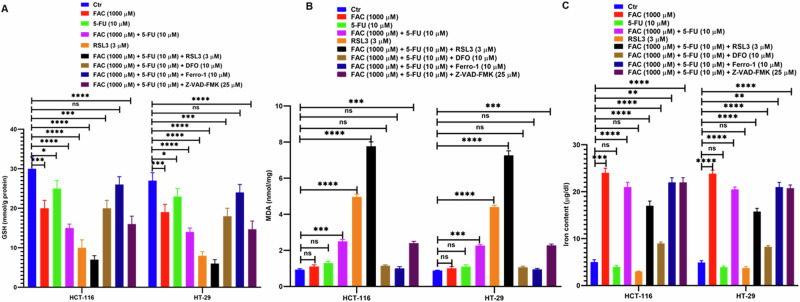
Determination of GSH, MDA and iron content in FAC and 5-FU combination treated CRC cells. Estimation of (**A**) GSH, (**B**) MDA, and (**C**) Iron content in HCT-116 and HT-29 cells exposed to FAC (1000 μM), 5-FU (10 μM), (FAC (1000 μM) + 5-FU (10 μM), RSL3, RSL3 + (FAC (1000 μM) + 5-FU (10 μM), DFO + (FAC (1000 μM) + 5-FU (10 μM), Ferro-1 + (FAC (1000 μM) + 5-FU (10 μM), Z-VAD-FMK + (FAC (1000 μM) + 5-FU (10 μM), and left untreated for 24 h. Data represents the mean value ± SE of at least three independent experiments. Significant change from the control is indicated by *p ≤ 0.05, **p ≤ 0.01, ***p ≤ 0.001 and ****p ≤ 0.0001

**Fig. 10:**
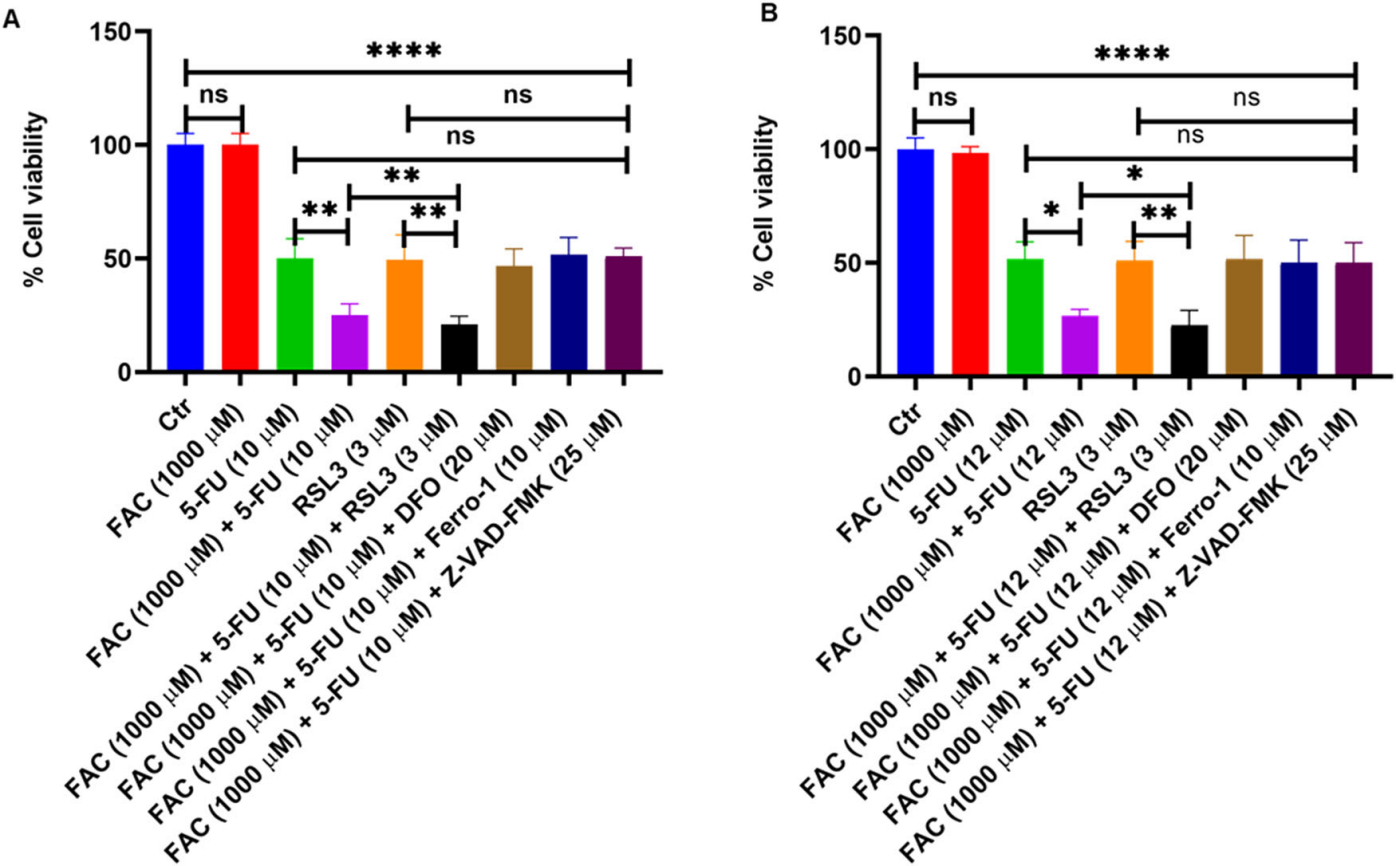
Cell viability of CRC cells exposed to ferroptosis and apoptosis inhibitors/activators in combination with FAC and 5-FU in HCT-116 and HT-29 cells. Cell viability determined by MTT assay in (**A**) HCT-116 and (**B**) HT-29 cells exposed with to FAC (1000 μM), 5-FU (10 μM), (FAC (1000 μM) + 5-FU (10 μM), RSL3, RSL3 + (FAC (1000 μM) + 5-FU (10 μM), DFO + (FAC (1000 μM) + 5-FU (10 μM), Ferro-1 + (FAC (1000 μM) + 5-FU (10 μM), Z-VAD-FMK + (FAC (1000 μM) + 5-FU (10 μM), and left untreated for 24 h. Data represents the mean value ± SE of at least three independent experiments. Significant change from the control is indicated by *p ≤ 0.05, **p ≤ 0.01 and ****p ≤ 0.0001

Furthermore, to verify the induction of apoptosis and ferroptosis in FAC pretreated/5-FU-treated CRC cells. After FAC pretreatment for 24 h, CRC cells (HCT-116 and HT-29) were treated with iron chelator deferoxamine (DFO; 10 µM), apoptosis inhibitor (Z-VAD-FMK; 25 µM) and activator/inhibitor of ferroptosis (RSL3; 3 µM/Ferro-1; 10 µM) before 5-FU treatment for next 24 h alone or in combination with FAC pretreated/5-FU-treated cells and untreated cells as control. As shown in Fig. 8, after treatment cell lysates were prepared to evaluate the quantification of apoptotic and ferroptosis-associated proteins. Our immunoblotting results revealed a decrease in the expression of antiapoptotic protein Bcl-xL with a subsequent increase in proapoptotic proteins Bak and Bax in cell lysates treated with 5-FU alone and FAC pretreated/5-FU-treated cells in both the cell lines (Fig. 8A–D). While cell lysates treated with RSL3 alone or in combination with FAC pretreated/5-FU-treatment, the expression of Bcl-xL is markedly decreased in HCT-116, and no significant change was observed in HT-29 cells. Intriguingly, we observe a marked decrease in protein expression of Bak and Bax in cell lysates treated with RSL3 and RSL3 in combination with FAC pretreated/5-FU-treated CRC cells relative to untreated cells. Moreover, we observe more or less the same protein expression of Bcl-xL, Bak, and Bax in cell lysates treated with DFO, Ferro-1, and Z-VAD-FMK compared to untreated control cells. To determine ferroptosis-associated GPX4 and TFR1 protein expression in the aforementioned treated cells, we perform immunoblotting. As shown in (Fig. 8A, E, F) a drastic decrease in GPX4 protein expression in 5-FU alone and FAC pretreated/5-FU-treated cells which was further decreased in the cell lysates treated with RSL3 alone or RSL3 in combination with FAC pretreated/5-FU-treated cells. However, we found a significant increase in GPX4 expression in cell lysates treated with DFO, Ferro-1, and Z-VAD-FMK in combination with FAC pretreated/5-FU-treated cells compared to untreated cells (Fig. 8A, F). Moreover, a marked decrease in expression of TFRC was observed in 5-FU alone whereas an increase in TFR1 protein expression was observed in FAC pretreated/5-FU-treated, RSL3, and RSL3 in combination with FAC pretreated/5-FU-treated cell lysates compared to untreated cells (Fig. 8A, E). However, in cell lysates exposed to DFO, Ferro-1, and Z-VAD-FMK in combination with FAC pretreated/5-FU-treated cells, the expression of TFRC is more or less the same as that of untreated cells. Furthermore, we also performed GSH, MDA and iron assay to determine antioxidant, lipid peroxidation and prooxidant status in FAC and 5-FU treated CRC cells in presence of ferroptosis activator RSL, ferroptosis inhibitor Ferro-1, iron chelator DFO and pancaspase inhibitor Z-VAD-FMK along with untreated control cells. As showed in (Fig. 9A) GSH levels significantly decreased in cells treated with FAC, 5-FU, treated CRC cells, however, the effect is more drastic in decreasing intracellular GSH levels in cell lysates treated with FAC, RSL-3 alone or RSL3 in combination with FAC pretreated/5-FU-treated cells. Moreover, we observe marked restorage of GSH levels in CRC cell lysates treated with DFO, and Ferro-1 in combination with FAC pretreated/5-FU-treated cells. Subsequently, intracellular MDA levels which depicts the lipid peroxidation levels was observed significantly more in FAC pretreated/5-FU-treated, RSL3, and RSL3 in combination with FAC pretreated/5-FU-treated cell lysates compared to FAC, or 5-FU alone and untreated cells (Fig. 9B). As shown in (Fig. 9C) iron content was significantly more in different groups of CRC cell lysates treated with FAC, however, CRC cell lysates from DFO in combination with FAC pretreated/5-FU-treated cells is less because of chelating effect of DFO. Additionally, we also determine the cell viability of the aforementioned treatments by trypan blue exclusion assay in CRC cells. As shown in Fig. 10A, B, percent cell viability is significantly decreased in 5-FU and RSL3 alone treated CRC cells which subsequently further reduced in 5-FU/RSL3 in combination with FAC pretreated/5-FU-treated cells compared to untreated control cells. While FAC pretreated/5-FU-treated cells exposed to DFO, Ferro-1, and Z-VAD-FMK rescue cell viability to around 50% compared to untreated control cells. Taken together, these findings suggest that FAC pretreated/5-FU-treated cells augment cell death via ferroptosis and apoptosis by downregulating and upregulating GPX4 and Bak/Bax expression, respectively in CRC cells. Moreover, decreased and increased expression of GSH and MDA levels, respectively, also supports induction of ferroptosis in treated CRC cells. The addition of ferroptosis or apoptosis inhibitors to FAC-pretreated/5-FU-treated CRC cells allows cells to rescue cell death either from ferroptosis or from apoptosis, respectively which eventually increases the percentage of cell viability of FAC-pretreated/5-FU treated CRC cells.

## Discussion

Currently, the drug of choice for CRC metastatic patients is 5-FU, which has been utilized in combination with oxaliplatin and folinic acid [17]. However, owing to the development of resistance, toxicity, and cost, the five-year survival remains dismal (less than 14%) [18]. Therefore, there is an urgent need for the development of novel therapeutic strategies to enhance the chemosensitivity of CRC to available chemotherapeutic agents. In the present study, we evaluated the chemosensitization potential of the food additive FAC in CRC. Our findings demonstrated that pretreatment with FAC induced oxidative stress as evidenced by ROS production and that FAC-pretreated/5-FU-treated CRC cells augmented cell death (apoptosis and ferroptosis) and promoted G2/M cell cycle arrest. Our results suggested that the FAC-pretreated/5-FU combination can sensitize CRC cells to the cytotoxic effects of 5-FU [19].

Besides being used as a food additive or fortifier in various baking and dairy products, FAC is used to supplement iron as a hematinic agent in iron deficiency anemia [3]. As is well known, cellular iron metabolism is strictly regulated at the uptake, usage, storage, and release stages [19]. In this context, existing evidence suggests that iron deficiency is a common finding in CRC and that it further promotes oncogenicity by way of diminishing immune surveillance in the tumor microenvironment [20]. Data from clinical studies also demonstrated that CRC patients with iron deficiency respond poorly to chemotherapy [21]. Gene expression data in colon cancer tissues showed that iron transport is impaired during colon tumorigenesis and that iron metabolism dysregulation in CRC patients is predictive of poor prognosis [20]. In that, increased cellular iron content was reported to enhance cell sensitivity to ferroptosis and to lead to immunogenic cell death by reinforcing host antitumor immunity [22]. Moreover, iron overload therapy was previously reported to improve the quality of life in CRC patients [23]. To understand how FAC treatment further disrupts cellular iron metabolism in CRC cells, the expression of key iron metabolism proteins was investigated following FAC treatment. Our analysis showed that the expression of FTH1, HMOX-1, and IRP-1 was elevated while that of hepcidin was reduced in FAC-pretreated/5-FU-treated cells. Such changes were either opposed to or more pronounced than the known pattern of expression of such genes in primary colon tumor samples.

Recent reports have demonstrated that accumulation of cellular iron not only governs various rate-limiting enzymatic reactions that support cellular growth, proliferation, and nucleic acid synthesis for newly proliferating cells [24], but also generates ROS species via the Fenton reaction thereby promoting oxidative stress, inflammation, and oncogenic signaling pathways that favor neoplastic growth [25]. However, excess iron, which induces severe oxidative stress, promotes lipid peroxidation and disrupts cell membrane integrity, eventually leading to ferroptosis [26]. Previous studies have also suggested that while FAC in different experimental settings induces antiproliferative effects [3], the exact mechanism underlying such effects is not well understood. Consistent with previous studies, our findings revealed that FAC exhibited negligible antiproliferative effects in HCT-116 and HT-29. However, FAC-pretreated/5-FU treatment drastically decreased cell viability when compared to 5-FU alone-treated or untreated cells. The antiproliferative effects of FAC-pretreatment/5-FU treatment in CRC cells are convincingly more than 5-FU alone treated cells. Additionally, we noticed more cell rounding and floating, indicating significant levels of cell death in FAC-pretreated/5-FU-treated cells compared to 5-FU or FAC alone treated or untreated cells after 24 h of treatment. Suggesting that, besides iron-overload, FAC increases the expression of intracellular iron metabolism-associated proteins, which later induce oxidative stress, and that upon treatment with 5-FU, FAC-pretreated/5-FU-treated cells undergo chemosensitization, thereby augmenting the significant antiproliferative effect than 5-FU alone treated cells.

The majority of antineoplastic drugs potentiate cell death by apoptosis [27], which involves the activation of caspases, shrinkage in cell size and membrane blebbing, nuclei condensation [28], and activation of PARP1 with simultaneous progression in internucleosomal degradation of DNA [22, 23]. Besides natural products, small molecule inhibitors in combination with 5-FU induce synergistic apoptotic cell death mechanisms, thereby achieving significant efficacy with less toxicity in vitro and in vivo models [29–31]. GDC-0326, a specific PI3K-α inhibitor was previously shown to promote the antiproliferative effect of 5-FU by activating necroptosis in CRC cells [32]. Consistent with previous findings, significant levels of apoptosis and necroptosis were observed in FAC-pretreated/5-FU-treated cells relative to FAC or 5-FU alone treated cells and untreated controls. Additionally, recently it was reported that treatment with 5-FU in conjunction with various natural products induces mitochondrial potential depolarization and mitochondria-dependent apoptotic cell death [33]. Such events may lead to further disruption of the stoichiometric ratio of mitochondrial resident anti-apoptotic (Bcl-2, Bcl-xL) and pro-apoptotic proteins (Bim, Bad, Bax) and the release of cytochrome C into the cytosol [34]. Consistent with previous observations, a significant increase in MMP depolarization as well as upregulation of mitochondrial resident apoptotic proteins Bak, Bax and downregulation of Bcl-xL expression in FAC-pretreated/5-FU-treated cells was consistently observed compared to untreated or FAC alone treated CRC cells [35].

Attenuation of the cell cycle at different phases is one of the strategies anticancer drugs employ to inhibit cell growth and induce cell death [30, 31]. Drugs targeting cell cycle-related proteins as a means of disrupting mitotic spindle formation are an appealing strategy to curtail malignancy. Previous work has shown that 5-FU alone or in combination with various natural products [36] and chemosensitizers [37], can attenuate cell cycling at different phases to inhibit CRC cell growth. Whereas normal cells utilize the G1 checkpoint to allow for DNA damage repair, malignant cells mostly rely on the G2 checkpoint to negate themselves from any DNA damage response [38]; the G2 checkpoint is an appealing target in anti-cancer drug discovery. Consistent with previous studies, our data showed that 5-FU alone treated as well as FAC-pretreated/5-FU-treated cells experienced a G2/M cell cycle arrest. It is well established that the interaction of cyclins with cyclin-dependent kinases is critically essential for cell cycle regulation [39]. Previous work has shown that 5-FU in combination with other molecules inhibits cyclin D1, and C-Myc, hence its clear effect on cell cycle progression [40] as reported here. Consistent with these reports, both FAC-pretreated/5-FU-treated cells, and 5-FU alone treated cells showed significantly reduced expression of cyclin D1, C-Myc, and survivin and significantly increased expression of cell cycle checkpoint inhibitors p21 and p27; these effects were more pronounced in FAC-pretreated/5-FU-treated cells.

Ferroptosis, which is commonly characterized by oxidative stress with aberrant ROS production, iron-dependent accumulation of lipid peroxidation, and deregulation of the antioxidant GSH system [41], is intricately involved in cancer biology [42]. GPX4, a master regulator of the intracellular antioxidant system, plays a critical role by negatively regulating ferroptosis [43]. Previous reports have shown that, in general, GPX4 expression is elevated in malignant cells perhaps as a means of balancing the increased accumulation of ROS and other forms of free radicals in malignant cells [44]. Recently, several compounds including ML162 [45], RSL3 [46], and DPI [47] were reported to inhibit GPX4 and GSH activity, and promotes MDA activity thereby inducing ROS-mediated ferroptosis induction in tumor cells. Additionally, Chang et al. reported that heteronemin, a marine terpenoid natural compound promotes ROS-mediated apoptosis and ferroptosis in hepatocellular carcinoma cells by downregulating GPX4 expression [48]. Consistent with previous reports, we observed a sharp increase in ROS and MDA production in FAC-treated and FAC-pretreated plus 5-FU-treated cells. Moreover, upregulation of proapoptotic proteins Bak and Bax with drastic reduction of antioxidant proteins GPX4 expression and GSH activity was significantly reduced in FAC-pretreated/5-FU-treated cells, no significant change was observed in GPX4 expression in FAC alone-treated cells.

While our study has provided comprehensive validation of our findings through several experiments, including in silico analysis of the TCGA dataset from patient samples and verification using two CRC cell lines, we acknowledge the limitation of not including animal model experiments. The in silico analysis allowed us to leverage a robust and extensive collection of genomic data from CRC patients, ensuring the relevance of our findings to human disease. However, we recognize that animal models are crucial for further validating the biological and translational relevance of our results, hence; will be incorporated in future studies.

In conclusion, the data presented here clearly suggest that FAC pretreatment sensitizes CRC cells to the cytotoxic effects of 5-FU. Mechanistically, 5-FU treatment in FAC-pretreated CRC cells induces significant ROS-mediated oxidative stress, G2/M cell cycle arrest, and ferroptosis. Further, in vivo studies are still needed to establish the tolerability of FAC treatment in cancer patients on chemotherapy.

## Supplementary information

Supplementary Information
