# Supplementary material for: Iron Overloading Potentiates the Antitumor Activity of 5-Fluorouracil by Promoting Apoptosis and Ferroptosis in Colorectal Cancer Cells
Source: Cell Biochem Biophys. 2024 Aug 4;82(4):3763–80. doi: 10.1007/s12013-024-01463-x (PMC11576816; doi:10.1007/s12013-024-01463-x)
Supplement: Supplementary file 1 — Supplementary Information [file 12013_2024_1463_MOESM1_ESM.docx]

**Supplementary Figures and Figure Legends**


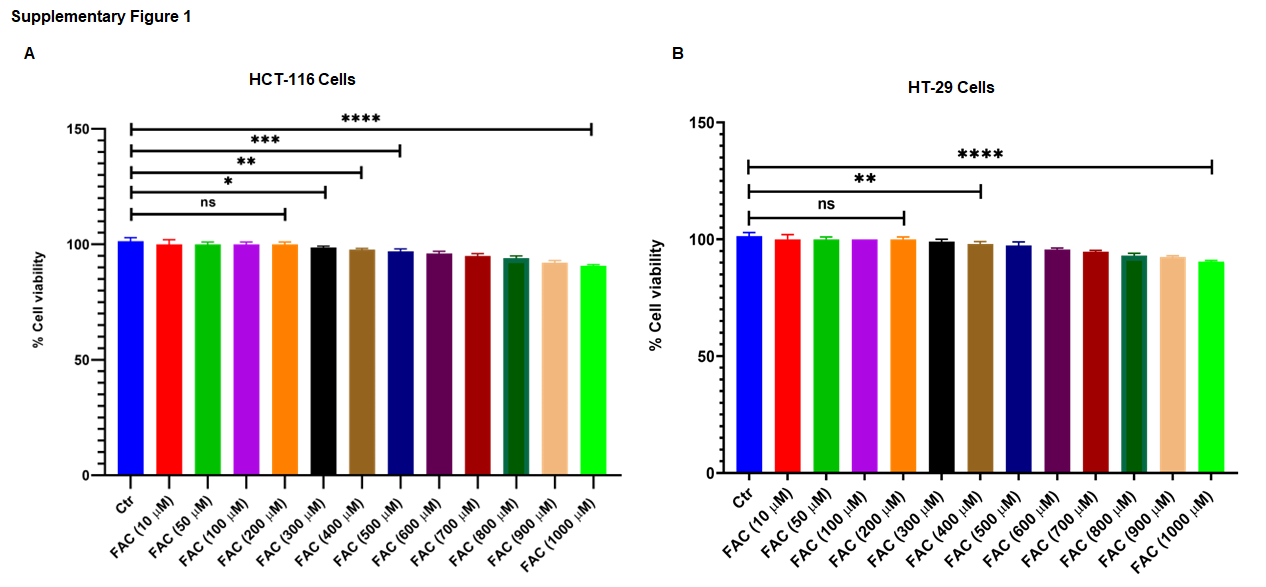


**Supplementary Figure 1. Cell viability of FAC treated HCT-116 and HT-29 cells.** Dose-dependent cell viability by trypan blue exclusion assay of (**A**) HCT-116 and (**B**) HT-29 cells, treated with varying doses of FAC (10, 50, 100, 200, 300, 400, 500, 600, 700, 800, 900, and 1000 μM) for 24 h. Data represent the mean value ± SE of at least three independent experiments. Significant change from the control is indicated by *p≤ 0.05, **p≤ 0.01 and ****p≤ 0.0001.


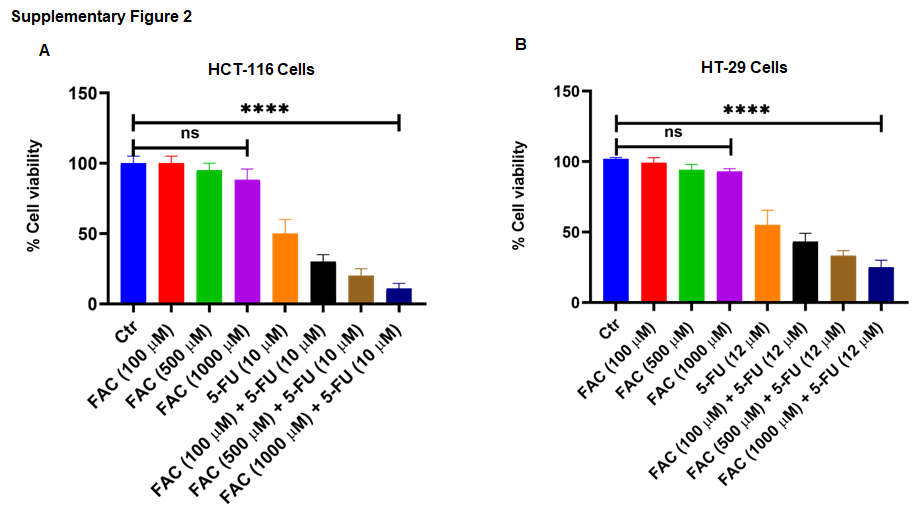


**Supplementary Figure 2. Cell viability of a combination of FAC and 5-FU in HCT-116 and HT-29 cells.** Cell viability determined by trypan blue exclusion assay in (**A**) HCT-116 and (**B**) HT-29 cells exposed with to FAC (100, 500, 1000 μM) alone, FAC (100, 500, 1000 μM) + 5-FU (10 μM), 5-FU (10μM) alone for 24 h. Data represents the mean value ± SE of at least three independent experiments. Significant change from the control is indicated by *p≤ 0.05, **p≤ 0.01 and ****p≤ 0.0001.
